# Throat culture positivity rate and antibiotic susceptibility pattern of *beta*-hemolytic streptococci in children on secondary prophylaxis for rheumatic heart disease

**DOI:** 10.1186/s12879-016-1841-3

**Published:** 2016-09-23

**Authors:** Nigus Zegeye, Daniel Asrat, Yimtubezinash Woldeamanuel, Abebe Habte, Etsegenet Gedlu, Tone Tønjum, Abraham Aseffa

**Affiliations:** 1 Department of Medicine, DebreBerhan University, P. O. Box: 445, DebreBerhan, Ethiopia; 2 Armauer Hansen Research Institute, Jimma Road, PO Box 1005, Addis Ababa, Ethiopia; 3 Department of Microbiology, Immunology and Parasitology, School of Medicine, College of Health Sciences, Addis Ababa University, Addis Ababa, Ethiopia; 4 Department of Pediatrics and Child Health, School of Medicine, College of Health Sciences, Addis Ababa University, Addis Ababa, Ethiopia; 5 Department of Microbiology, Oslo University Hospital, Oslo, Norway; 6 Department of Microbiology, University of Oslo, Oslo, Norway

**Keywords:** *Beta* hemolytic streptococci (BHS), Rheumatic heart disease (RHD), Rheumatic fever (RF), Antibiotic prophylaxis, *Streptococcus pyogenes*, *Streptococcus dysgalactiae subsp. equisimilis*, Antibiotic resistance, Ethiopia

## Abstract

**Background:**

Among children diagnosed to have chronic rheumatic valvular heart disease (RHD) in Ethiopia, many have been observed to develop recurrence of rheumatic fever (RF) despite secondary prophylaxis. This study determined the throat culture positivity rate and drug susceptibility pattern of *beta* hemolytic streptococci (BHS) isolated from children attending a specialized cardiac clinic in Ethiopia.

**Methods:**

Throat swabs were collected from 233 children receiving benzathine penicillin injection as secondary prophylaxis for RHD and cultured. The bacterial isolates were characterized using Matrix Assisted Laser Desorption/Ionization-Time of Flight (MALDI-TOF) mass spectrometry. Drug susceptibility was tested with the Kirby Bauer disc diffusion method. Anti-streptolysin O (ASO) titers were determined using ASO latex reagents.

**Results:**

The throat culture positivity rate for BHS was 24 % (56/233). Among the BHS bacterial strains isolated, four were characterized as *S. pyogenes* and another four as *S. dysgalactiae subsp. equisimilis* (Lancefield group A, C and G). All BHS were susceptible to penicillin except one isolate of *S. agalactiae*. Among 233 children enrolled, 46(19.7 %) showed increased ASO titer. Children who received antibiotic prophylaxis within 2-weeks of last injection had significantly lower BHS throat culture positivity rate than those injected every 4-weeks (*p* = 0.02). Children who missed at least one prophylaxis within the last 6 months had a higher BHS culture positivity rate than those who did not miss any (*p* = 0.0003).

**Conclusions:**

The presence of groups A, C and G streptococci in the throat of children under secondary prophylaxis for RHD and increased ASO titer suggests failure of the regimen. This calls for further investigation into the causes of inadequate prophylaxis (including bioavailability of drugs used, optimal duration and patient compliance) and intervention.

## Background

Rheumatic fever (RF) is a non-suppurative, auto-inflammatory multi-system response following infection by group A *beta* hemolytic streptococci (BHS) also known as *Streptococcus pyogenes* [1]. *Streptococcus dysgalactiae subsp. equisimilis* expressing Lancefield group A (GAS), C (GCS) or G (GGS) antigen is phylogenetically related to *S. pyogenes.* It has recently emerged as a potential pathogen and there is concern that it might cause human infections similar to *S. pyogenes* including RF [2–5]. Eleven non-group A-BHS strains have previously been recovered from RF and/or rheumatic valvular heart disease (RHD) patients in Ethiopia [6].

Rheumatic heart disease is the most dreaded complication of recurrent RF. Unless treated, the valve damage can eventually lead to chronic intractable heart failure and premature death. In developing countries, facilities for this treatment are almost non-existent [7]. Although its incidence is decreasing in industrialized countries, RHD remains a major challenge in the rest of the world. The highest prevalence is in sub-Saharan Africa with a rate of 5.7 per 1000, compared to 1.8 per 1000 in North Africa, and 0.3 per 1000 in economically advanced countries [8]. In Ethiopia, RHD is the number one cardiac problem in children with a prevalence rate of 4.6–7.1 per 1000 [9, 10]. Data from hospitals indicate that about one third [32.8 % (256/781) [11] and 39.2 % (127/324) [12]] of heart disease cases are due to RHD. Among 457 cardiovascular deaths including cerebrovascular accidents (CVA) from January 1995 to December 2001 in the current study site, TikurAnbessa Specialized Hospital (TASH), 26.5 % (121) were due to RHD [13]. Günther*et al.* also reported 125.3 per 1000 person-year mortality rate during 7 years of follow up of RHD patients at Dabat Health Centre in North Gondar, which amounted to 12.5 % annual mortality rate among RHD patients seen in this community [14].

The economic effects of the disability and premature death caused by these diseases are felt at both the individual and national levels through increased direct and indirect health care costs. The most cost effective approach for the control of RHD is secondary prophylaxis with penicillin injection every 3 or 4-weeks [14]. Careful penicillin delivery results in recurrence prevention also in high risk areas [15]. However, in our study setting in Ethiopia, implementation of secondary antibiotic prevention is challenged by missed opportunities for treatment, poor access to health care and inadequate treatment of tonsillopharyngitis with failure to eradicate *S. pyogenes* from the throat [14, 16]. Therefore, we investigated the throat culture positivity rate, BHS strain types and antibiotic susceptibility patterns and their relations to dose intervals of penicillin in children on secondary prophylaxis for RHD at TASH.

## Methods

### Study design and population

Two hundred and thirty-three children who were on secondary prophylaxis for RHD were enrolled in the study. The participants were recruited consecutively as they came to the cardiac clinic for their follow up appointment. The study was conducted at the Pediatric Cardiac Clinic of TASH in Addis Ababa, Ethiopia, between July 2013 and June 2014.

Ethical clearance was obtained from the Ethics Review Committees of the College of Health Sciences, Addis Ababa University and the Armauer Hansen Research Institute/All African Leprosy Rehabilitation and Training Centre (AHRI/ALERT). Written informed consent was obtained from each child’s parent or guardian and assent from study participants older than 12 years. Parents/ guardians consented to sending bacterial isolates for typing at the Oslo University Hospital, University of Oslo, Norway. Sample export permission was obtained from the Ethiopian Institute of Biodiversity and the Oslo University Hospital, University of Oslo, approved the analysis of the bacterial isolates.

### Socio-demographic, risk factors, clinical and laboratory data

General socio-demographic characteristics and risk factors for the spread of BHS including assessment of adherence to penicillin intake were collected by a structured questionnaire. Additional laboratory and clinical data were collected from patient records.

### Sample collection and processing

Throat swab samples were collected by trained nurses. The tonsillar fauces and the posterior pharyngeal wall behind the uvula were swabbed using sterile swab applicators (Thermo Fisher Scientific, USA). Each swab was immersed into a test tube containing skim milk-tryptone-glucose-glycerin (STGG) medium (Thermo Fisher Scientific, USA). The samples were stored at +4 °C and transported to the AHRI bacteriology laboratory within 8 h. Two ml of venous blood was aseptically collected from each study participant and serum stored at -80 °C. Anti-streptolysin O (ASO) latex reagents (LiNEAR Chemicals. s. L, Spain) were used to determine ASO titer according to manufacturer’s recommendations.

### Isolation and characterization of ß-hemolytic streptococci

The throat swabs were streaked on 7 % defibrinated sheep blood agar plates (Becton, Dickinson, USA) and incubated under CO_2_ at 37 °C for 24–48 h. ß*-*hemolytic colonies were subcultured, and the isolates were tested with catalase test, Gram stain, and bacitracin susceptibility test. All catalase-negative Gram-positive cocci were stored in STGG media at -80 °C and transported to the University of Oslo on dry ice for further characterization. All BHS isolates were serogrouped by a streptococcal grouping latex kit (Pro-Lab Diagnostics, USA) according to the manufacturer’s instructions.

Matrix-assisted laser desorption/ionization-time of flight (MALDI-TOF) mass spectrometry (MS) analyses was performed at the University of Oslo by the direct colony method by using the Microflex mass spectrometer (BrukerDaltonics, Germany) for species differentiation. Streptococcal strains isolated from throat swabs were re-cultivated on 5 % human blood agar media. A 1 μL inoculation loop full of each of the bacterial isolates was obtained from a fresh culture, deposited in duplicate on the target plate and smeared. The bacterial film was first overlaid with 1 μL formic acid (100 %) and then with 1 μL matrix α-cyano-4-hydroxycinnamic acid (HCCA). Two spots were prepared for each bacterial isolate. The target plate was inserted into the Microflex mass spectrometer and the spectra generated were analyzed by the MALDI Biotyper 3.0 software (BrukerDaltonics, Germany) and matched to the Biotyper 3.0 database.

All BHS were tested for susceptibility to commonly used antibiotics with the disk diffusion method on Mueller Hinton Agar (MHA) (Thermo Fisher Scientific, USA) supplemented with 5 % defibrinated sheep blood using standard methods. The BHS were tested against the following antibiotics: penicillin G (10 units), oxacillin (1 μg,), ceftriaxone (30 μg), vancomycin (30 μg), erythromycin (15 μg), tetracycline (30 μg), ofloxacin (5 μg), chloramphenicol (30 μg), clindamycin (2 μg), quinopristin-dalfopristin (15 μg), linezolid (30 μg), and trimethoprim-sulfamethoxazole (25 μg). Test interpretation was done according to the Clinical Laboratory Standards Institute (CLSI) [17].

### Data management and analysis

Data was entered and summarized using SPSS version 20 software (USA) and analyzed using the STATA software (*StataCorp* LP, College. Station, Texas, USA). Comparisons were made using the Chi—square test. A *p*-value of ≤ 0.05 was considered indicative of a statistically significant difference.

## Results

### Socio-demographic characteristics of the study participants

Two hundred and thirty-three children in the age range of 5–15 years who were on secondary prophylaxis for RHD participated in this study. Three-fourths of them were aged between 10 and 15 years and girls accounted for 59.1 %. All of the participants were on monthly penicillin G prophylaxis. The most common valve lesions observed in the children were mitral regurgitation (92.3 %), tricuspid regurgitation (67.0 %) and aortic regurgitation (54.1 %). Eighty-nine (38.2 %) participants had been referred for possible surgical interventions, but only three had received this treatment.

### Frequency of *beta-*hemolytic streptococcal culture positivity in children under prophylaxis

A total of 58 BHS were isolated from fifty-six participants. The throat culture positivity rate of BHS was 24 % (56/233). GAS occurred in 2.6 % (6/233) and accounted for 10.3 % of the BHS (6/58). Twenty-five (43.1 %) of the BHS belonged to serogroup F, 22 (37.9 %) to serogroup G, 2 to serogroup C, 1 to group B and 2 could not be allocated to any type. In addition to conventional characterization, all streptococcal strains were identified to the species level by MALDI-TOF MS (Table 1). The majority of the isolates belonged to *S. anginosus* (36.2 %) and *S. constellatus* (43.1 %). *S. pyogenes and S. dysgalactiae subsp. equisimilis* accounted for 6.9 % each, while 5.2 % could not be classified. Among the 6 group GAS isolates, four were bacitracin-susceptible *S. pyogenes* and the other 2 were bacitracin-resistant *S. dysgalactiae subsp. equisimilis. S. dysgalactiae subsp. equisimilis* strains expressing either Lancefield group A, C or G were detected in this study.

**Table 1:** Species and serogroup distribution of *beta* hemolytic streptococci isolated from children with rheumatic heart disease

Species	Serogroup	No	%
*S. pyogenes*	A	4	6.9
*S. dysgalactiae subsp. equisimilis*	A	2	3.4
	C	1	1.7
	G	1	1.7
*S. agalactiae*	B	1	1.7
*S. anginosus*	G	20	34.5
	non-typeable	1	1.7
*S. constellatus*	F	24	41.2
	non-typeable	1	1.7
Not differentiated	C	1	1.7
	F	1	1.7
	G	1	1.7
Total		58	100

Speciation of 41 isolates which were difficult to distinguish as either *α* or *β* hemolytic on sheep blood agar plates showed that the majority were either *S. parasanguinis* or *S. mitis* (31.7 % each). *S. oralis, S. pneumoniae, S. constellatus, and S. perioris* accounted for 7.3, 4.9, 2.4, and 2.4 % of these strains, respectively. However, 19.5 % bacitracin-susceptible isolates could not be classified further. Most *S. parasanguinis* (84.6 %) and about half of *S. mitis* (54.5 %) were similarly bacitracin-sensitive. One single *a*-hemolytic *S. constellatus* isolate was found to possess Lancefield group C antigen.

### Antibiotic susceptibility

All BHS isolates were susceptible to penicillin except for one strain of *S. agalactiae.* This isolate was also resistant to tetracycline, oxacillin, ceftriaxone, and vancomycin. All four *S. pyogenes* isolates were susceptible to penicillin and vancomycin. One isolate of *S. pyogenes* was resistant to erythromycin and another intermediately resistant. Overall, the sensitivity of BHS to erythromycin was 87 % (48/55 tested). All BHS were non-susceptible to oxacillin, and almost half of them (46.3 %) were also non-susceptible to ceftriaxone.

### Anti-streptolysin O titer

**Fig. 1:**
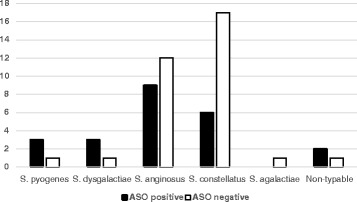
Distribution of BHS species and ASO titer among children on secondary antibiotic prophylaxis for RHD

**Fig. 2:**
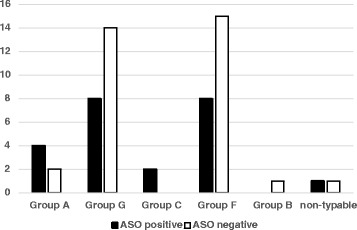
ASO titer and BHS serogoup distribution among children on secondary antibiotic prophylaxis for RHD

Among 233 participants, 69 (29.6 %) had a positive ASO titer (≥200 IU/ml). Of these, 66.7 % (46/69) had ASO > 200 IU/ml, suggesting recent infection. The prevalence of an increased ASO titer was 19.7 %. Out of 8 children who had *S. pyogenes* or *S. dysgalactiae* in their throat culture, 6 had ASO titer ≥ 200 IU/ml, indicating an active infection. There was a statistically significant association between BHS culture positivity and ASO positivity (*p* = 0.0315) (Table 2). Among 56 patients for whom species and serogroups could be determined, 23 were positive for ASO titer (Figs. 1 and 2).

**Table 2:** Relationship between ASO titer and BHS infection in children on antibiotic prophylaxis for RHD

	ASO (titer ≥200 IU/ml)			*P*-value
	Positive	Negative	Total	
BHS isolated	23	33	56	0.0315
BHS negative	46	131	177	
Total	69	165	233

### Relationship between isolation of BHS from the throat and antibiotic use

Participants missing prophylaxis had a higher likelihood of having BHS in their throat (*p* = 0. 0003) (Table 3). Children who received antibiotic prophylaxis at intervals of between 22 and 28 days had 1.6 times higher risk to have been colonized by BHS than those receiving prophylaxis at intervals of ≤16 days (*p* = 0. 02, OR = 2.6, CI =1. 16–5.85) (Table 4). Children who received antibiotic prophylaxis at an interval of ≥ 29 days had a 2.6 times higher risk of BHS colonization (*p* = 0.003, OR = 3. 61, CI = 1.5–8.46).

**Table 3:** Risk factors associated with BHS colonization of throat in children on antibiotic prophylaxis for RHD

Risk factors		*Beta-*hemolytic streptococci		
		Culture positive	Culture negative	*P*-value
Age^a^	5–9 (*n* = 55)	13	42	0.4303
	10–15 (*n* = 172)	40	132	
Sex^a^	Male (*n* = 95)	26	69	0.2758
	Female (*n* =137)	29	108	
Residence	Urban (*n* = 121)	27	94	0.6302
	Rural (*n* = 112)	28	84	
Family size	<6 (*n* = 139)	32	107	0.7990
	≥7 (*n* = 94)	23	71	
Missing at least one prophylaxis within last 6 months	Missing (*n* = 33)	16	17	0.0003
	Not missing (*n* = 200)	39	161	
Family history of pharyngitis	Yes (*n* = 25)	5	20	0.7235
	No (*n* = 203)	47	156	
Family educational status^a^	Illiterate (*n* = 68)	18	50	0.4739
	Literate (*n* = 163)	36	127	
Uvulectomy^a^	Yes (*n* = 57)	16	41	0.3566
	No (*n* = 159)	35	124	

^a^Missed values were excluded from analysis

**Table 4:** Duration of interval between penicillin injections as risk factor for throat colonization with BHS

Interval between antibiotic prophylaxis	Culture positive	Culture negative	OR (95 % CI)	*p*-value
<16 days (*n* = 98)	15	83	1	
17–21 days (*n* = 25)	5	20	1.4(0.5–4.3)	0.57
22–28 days (*n* = 50)	16	34	2.6(1.2–5.9)	0.02
≥29 days (*n* = 38)	15	23	3.6(1.5–8.5)	0.003

*CI* confidence interval, *OR* odds ratio

Of three participants who had *S. pyogenes*, two of them had received their last dose more than 16 days before but one had the last injection less than 16 days ago. In the same way, among 3 participants who had *S. dysgalactiae subsp. equisimilis*, two had received injection at an interval of more than 16 days and only one less than 16 days ago*.*

## Discussion

This study was carried out to determine the effectiveness of secondary antibiotic prophylaxis in eliminating GAS and other BHS from the throat of children with RHD under follow-up at a pediatric cardiac clinic of a tertiary hospital. Almost one quarter of the children (24 %) were culture positive for BHS in their throat, and 20 % had increased ASO titer suggesting active infection. Children who received antibiotic prophylaxis within 2-weeks of last injection had significantly lower BHS culture positivity rate than those injected every 4-weeks (*p* = 0. 02). Children who missed at least one prophylaxis injection within the last 6 months had a higher BHS culture positivity rate than those who did not miss any (*p* = 0.0003).

Defaulting from regular follow up is a strong contributor to mortality from RHD [14]. Four-week intervals of injections would be expected to have less defaulting rates than 3-week intervals. On the other hand, two-weekly and three-weekly intramuscular penicillin injections have been reported to perform better in reducing the recurrence of streptococcal throat infections and episodes of RF with higher concentration of penicillin in the serum when compared to four-weekly injection [18, 19]. The World Health Organization (WHO) and the American Heart Association both recommend a three-weekly regimen for individuals living in a high risk area [20, 21]. Although Ethiopia is a high risk area for RHD, the current regimen for secondary antibiotic prophylaxis in Ethiopia is a four- weekly administration of intramuscular penicillin G injections.

Among 230 patients, 30.9 % had a history of documented recurrence of RF which is comparable to a study conducted in a Pacific island population (38. of 144 participants) (*p* = 0. 145) [22]. Rheumatic heart disease frequency is higher in females (59.1 %), which is in agreement with population and hospital based studies conducted in Ethiopia and elsewhere [6, 23]. Eighty-nine (38.2 %) were referred for possible surgical intervention of which only three participants received this intervention. A delay in surgical intervention for more than 1 year was observed in 46.5 % (40/86) of cases. This delay of heart valve surgical intervention for more than 1 year was comparable with what it was like in Egypt 15-years ago [23].

In the current study, 29.6 % of the participants were positive for ASO and 20 % showed increased ASO titer indicating recent streptococcal infections. There was a statistically significant association between BHS culture positivity rate and ASO positivity, also considering patient age [24]. BHS expressing Lancefield group A, C and G infections are reported to cause a rise in ASO titer in serum [25–27]. This argues for infection rather than simple colonization.

The reason for a high prevalence of BHS and increased ASO titer despite antibiotic prophylaxis in the current study is not explained by lack of susceptibility to penicillin since no isolate was resistant to the drug in vitro. It appears that the timing of the antibiotic prophylaxis is a more significant factor. Children who received antibiotic prophylaxis within 2-weeks of the last dose exhibited significantly lower BHS culture positivity than those who received four-weekly injection (*p* = 0. 02). There was no statistically significant difference between 2- and 3-weeks of injection although a trend could be observed in that a higher percentage of participants showed more BHS culture positivity rate in the 3-week interval group. We have not evaluated the dynamics of penicillin bioavailability in our patients. Reduced serum penicillin G due to any cause may lead to ineffective prophylaxis. This could be due to poor quality of drugs as brands of penicillin prescribed may vary in effectiveness [23], but it could also be due to patient-specific factors. Some individuals have been reported to degrade penicillin G in their serum faster than others [18, 28]. This is obviously aggravated when the interval of injection is longer.

Recurrence of RF and reduced ability of penicillin to eradicate BHS are reported continuously from several parts of the world, indicating failure of benzathine penicillin G prophylaxis [18, 19, 29, 30]. A combination of factors has to be considered in each particular setting before an appropriate regimen is selected for the respective population [19]. One major problem in secondary prophylaxis for RHD is compliance. In our series, 14.2 % of the participants had missed one or more injections within the last 6-months of follow up, and these participants had a significantly higher BHS culture positivity rate (*p* = 0.0003) than others. We did not find any significant association of factors such as age, sex, history of family pharyngitis, uvulectomy and family educational status with culture positivity of BHS or failure of regimen.

The BHS culture positivity rate (24.0 %) was similar to a study conducted on chronic RHD patients about 25-years ago (20.45 %) (*p* = 0. 61) [6]. Although the children had received on-going monthly penicillin G prophylaxis this culture positivity rate was significantly higher than that reported for healthy schoolchildren in Addis Ababa by Abdissa and colleagues (17.7 %) (*P* = 0. 03) [31]. It seems that Group F and G BHS were prominent in the present study, a finding which is comparable with other studies conducted elsewhere, such as in Mumbai where Group C and G streptococcal disease burden was higher than GAS among schoolchildren [2, 32]. Even though the type of prophylaxis and the methodology is different, the present finding showed a lower culture positivity rate as compared with a carrier rate of 15.4 % GAS among 26 patients on once-weekly azithromycin (AZT), but higher than 22 patients taking oral penicillin (0 %) [33].

In addition to traditional methods (*beta*-hemolytic characteristics, catalase test, gram stains and serogouping), species identification was also performed by MALDI-TOF MS. Among six GAS, four were *S. pyogenes*. The other 2 were bacitracin-resistant *S. dysgalactiae subsp. equisimilis*. In the current study, *S. dysgalactiae subsp. equisimilis* possessing Lancefield group A, C and G were isolated. There are reports that *S. dysgalactiae subsp. equisimilis* expressing Lancefield group A is on the rise as a pathogen [3, 34]. In the present study, four *S. dysgalactiae subsp. equisimilis* were isolated from four RHD patients, raising a suspicion whether these organisms are associated with RHD. Pharyngeal Group C and G have often been detected in patients with RF/RHD in endemic countries but there is as yet no conclusive evidence whether these organisms do cause acute RF as is known for Group A strains [2, 6, 35, 36].

We observed that 3 BHS isolates and some *alpha-*hemolytic streptococcal isolates including *S. pneumoniae* were not correctly identified by MALDI-TOF MS speciation (very small score value and inconsistent results). Others have also observed that some strains were not correctly identified by this method and that improvements to this technique as well as complementary tests are needed [37].

In the present study, all GAS isolates were susceptible to penicillin, which is comparable with other studies conducted in Ethiopia and elsewhere [31, 38]. Penicillin-resistant GAS is not yet reported from this setting [38, 39]. Penicillin non-susceptible *S. agalactiae*/ group B streptococcus and susceptible group G and F streptococci were, however, observed in the present study, which is in contrast with studies conducted elsewhere [31, 32]. Penicillin and oxacillin susceptibility results were also discordant in this study.

In the present study, one GAS strain was erythromycin-resistant and another one intermediately susceptible to erythromycin. Erythromycin-resistant GAS strains are emerging. Studies have reported proportions of 96.1 % [39] and 38 % of GAS resistance to erythromycin [40]. One report high-lighted how macrolide treatment failure in streptococcal pharyngitis resulted in acute RF [40]. Thus, these are indications that antimicrobial drug resistance is emerging among BHS as well [32, 38]. For diagnostic purposes, the emerging bacitracin resistance *S. pyogenes* should be noted [41].

Presumptive identification of BHS on blood agar is not necessarily straight-forward. Human blood agar is not recommended for BHS isolation [42, 43]. We observed, as others have also experienced, that some *alpha-*hemolytic streptococci are difficult to differentiate from BHS, especially when the incubation time is extended to 48 h. Many of them were bacitracin susceptible (84.6 % of *S. parasanguinis*, 54.5 % of *S. mitis* and 8 unclassified others). In other studies, bacitracin susceptibility was reported in some GCS and as high as 67 % among GGS [44] and 12.2 % among non-group A BHS [45]. On the other hand, bacitracin resistance *S. pyogenes* was reported elsewhere [41]. This calls for improvements in typing techniques in clinical laboratories in order to monitor trends of RHD epidemiology and control.

## Conclusion

A relatively high prevalence of BHS was detected among RHD patients who were under secondary antibiotic prophylaxis. *S. pyogenes* and *S. dysgalactiae subsp. equisimilis* were isolated from the throat together with Group F and Group G streptococci. Therefore, the presence of GAS, GCS and GGS and increased ASO titer indicating recent streptococcal infection might suggest inadequate prophylaxis and may call for a review of the regimen in use and a better monitoring of trends with larger studies including bioavailability of penicillin lots in use. Continuous health education to caretakers and children regarding the importance of taking the benzathine penicillin prophylaxis on time as prescribed is recommended.

## Abbreviations

AHRI: Armauer Hansen Research Institute
ASO: Anti-Streptolysin O
BHS: *Beta* hemolytic streptococci
GAS: Group A Streptococcus
GCS: Group C streptococcus
GGS: Group G streptococcus
MALDI-TOF MS: Matrix Assisted Laser Desorption Ionization/-Time of Flight Mass Spectrometry
RF: Rheumatic Fever
RHD: Rheumatic Heart Disease
STGG: Skim milk, Trypton Soya broth, Glucose, Glycerin
TASH: TikurAnbessa Specialized Hospital
WHO: World Health Organization
